# Paraganglioma presenting as hypertension during pregnancy, proteinuria, thrombocytosis, and diabetes mellitus: a case report

**DOI:** 10.1186/s13256-021-02923-1

**Published:** 2021-07-09

**Authors:** Ramjan Sanas Mohamed, Charles Naveenan Antonypillai, Harishanthi Mahendran

**Affiliations:** Department of Diabetes and Endocrinology, Teaching Hospital Kandy, 379/3, Waragashinna, Akurana, Kandy, Sri Lanka

**Keywords:** Paraganglioma, Pregnancy-induced hypertension, Thrombocytosis, Diabetes mellitus

## Abstract

**Background:**

Paraganglioma is a very rare cause of pregnancy-induced hypertension. The objective of this case report is to present a case of paraganglioma presented during pregnancy and missed. Later, the diagnosis was made during the postpartum period because of persistence of hypertension.

**Case presentation:**

Here, we describe the case of a patient with paraganglioma who initially presented with pregnancy-induced hypertension and gestational diabetes mellitus. She had persistence of hypertension and diabetes mellitus following delivery with proteinuria, thrombocytosis, and spells. Once her pelvic paraganglioma was removed, her blood pressure and blood sugar were normal without antihypertensives or hypoglycemic agents, respectively. Her proteinuria settled with near-normal platelet counts.

**Conclusion:**

Although neuroendocrine tumors are a rare cause of pregnancy-induced hypertension, it should be suspected in the appropriate clinical setting. Diabetes mellitus, proteinuria, and thrombocytosis can be a clinical feature in paraganglioma.

## Introduction

A paraganglioma is a tumor derived from extraadrenal chromaffin cells of the sympathetic paravertebral ganglia of thorax, abdomen, and pelvis and parasympathetic ganglia located along the glossopharyngeal and vagal nerves in the neck and at the base of the skull. Paragangliomas are rare tumors [1]. The majority of sympathetic paragangliomas arise in the abdomen (75%). Hypertension is a common medical disorder in pregnancy. It may predate or arise *de novo* during pregnancy. Endocrine disorders are a rare cause of hypertension in pregnancy [2]. Pregnancies complicated by a pheochromocytoma or paraganglioma are very rare, estimated to occur in 0.007% of all pregnancies [2, 3]. Here, we describe the case of secondary hypertension during pregnancy, diabetes mellitus, proteinuria, and thrombocytosis due to pelvic paraganglioma.

## Case presentation

**Fig. 1:**
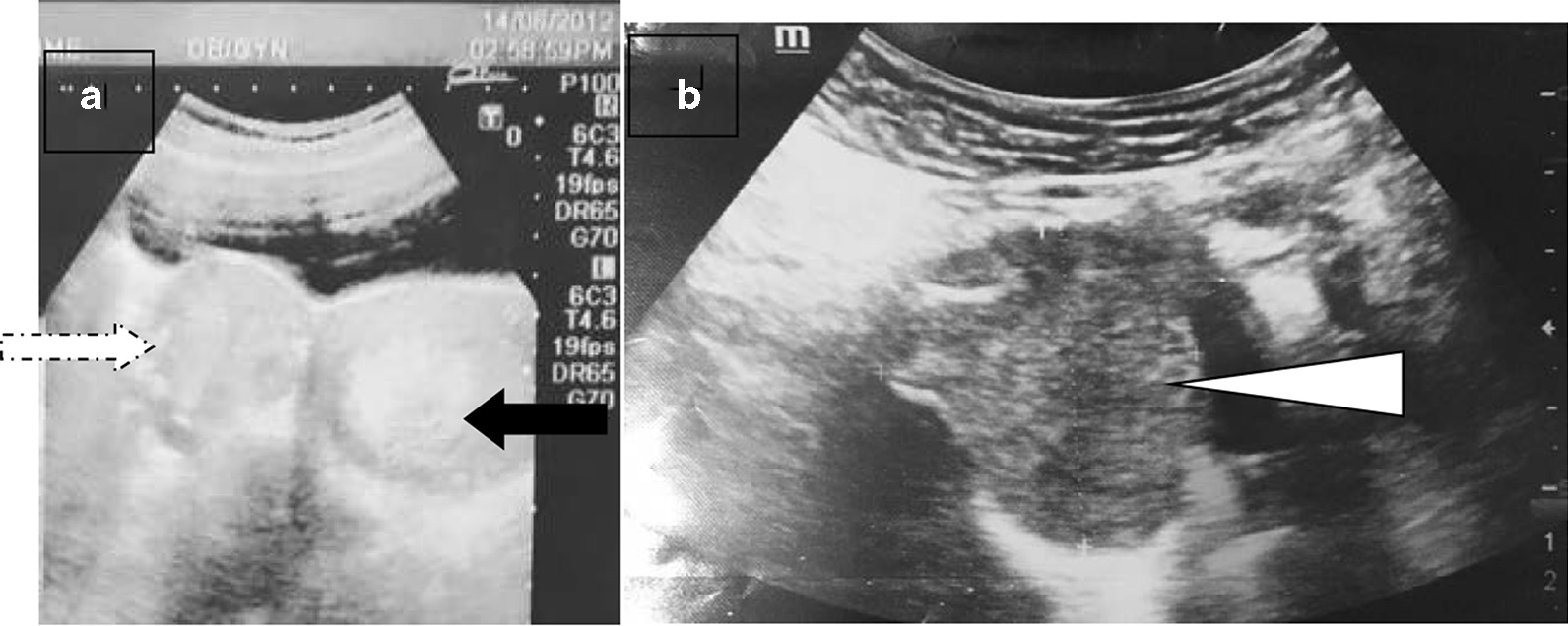
**A** Dotted arrow pelvic mass measuring 4.8 × 4.6 cm; black arrow indicates gestational sac. **B** Arrow indicates well-defined, hypoechoic lesion at fundus of the uterus measuring 5.5 × 4.5 cm with significant vascularity in 2017

A 24-year-old mother of two was referred to us with hypertension and diabetes mellitus for 18 months duration in 2017. During her first pregnancy, she had developed gestational diabetes mellitus (GDM) and hypertension in 2012. During the postpartum period, her hypertension and GDM had resolved. At her second pregnancy, at period of amenorrhea (POA) of 6 weeks, diabetes was identified, and she was started on insulin. During her second trimester, she again developed elevated blood pressure. Initially, her blood pressure (BP) was controlled with nifedipine, and at POA of 31 weeks, her BP was 110/70 mmHg. However, at this time, she was started on labetalol, developed uncontrolled BP (170/100 mmHg), and had to undergo emergency caesarean section at POA of 33 weeks, delivering a healthy baby without any complications. Following postpartum, she had persistently elevated BP and blood sugar. There were no features suggestive of diabetic microvascular or macrovascular complications. Her blood sugar was controlled with premixed insulin. She was on three antihypertensives: enalapril, hydrochlorothiazide, and nifedipine. She had been investigated for elevated platelets previously with several blood investigations including antinuclear antibody (ANA), which was negative. During the dating scan of her first pregnancy, a pelvic mass measuring 4.8 × 4.6 cm was found and was thought to be a pedunculated fibroid or corpus luteum in 2012 (Fig. 1A). Repeat ultrasound scan (USS) during postpartum did not reveal the mass. There was no known family history of young hypertension, pituitary surgeries, or calcium or thyroid problems.

Her body mass index was 20 kg/m^2^. She did not have features suggestive of Cushing’s syndrome or acromegaly. There were no acanthosis nigricans, skin nodules, hyper- or hypopigmented macules, or any neuromas. All her peripheral pulses were present with no radio radial or radio femoral delay. Her BP in both arms was 110/70 mmHg without postural drop. She did not have any murmurs. Her abdominal examination did not reveal any organomegaly or ballotable masses. She did not have renal bruit. Her fundal examination showed silver wiring, but there were no hemorrhages, papilloedema, or diabetic retinopathy. She did not have any focal neurological deficits. Her diabetes was controlled with premixed insulin.

## Investigations

Her 24-hour urinary metanephrine showed 1.3 mg/24 hours (<1 mg/24 hours), and chromogranin A level was 222.7 ng/ml (<98.1). Plasma or urinary fractionated metanephrines were not done because of unavailability. Her other investigations revealed hemoglobin 12.7 g/dl, white blood cell count (WCC) 9.9 × 103 µl, platelets 608 × 10^3^ µl, sodium 140 mmol/l, potassium 4 mmol/l, calcium 10.5 mg/dl, thyroid-stimulating hormone (TSH) 2.1 mIU/l, serum creatinine 0.5 mg/dl [estimated glomerular filtration rate (eGFR) 135 ml/min], urine full report (UFR) protein +, urine microalbumin/creatinine 133.62 mg/g, and HbA1c 8.5% (Table 1). Her blood picture revealed only thrombocytosis. Her repeat ANA was negative.

**Table 1:** Hematological, biochemical, and urinary investigations

Investigation	Results	Reference range
24-hour urinary metanephrine	1.3 mg/24 hours	<1
Chromogranin A level	222.7 ng/ml	<98.1
Hemoglobin	12.7 g/dl	
White cell count	9.9 × 10^3^ µl	4–11
Platelet	608 × 10^3^ µl	150–400
Sodium	140 mmol/l	135–145
Potassium	4 mmol/l	3.5–5
Calcium	10.5 mg/dl	8.1–11
TSH	2.1 miu/l	0.465–4.68
Serum creatinine	0.5 mg/dl (eGFR135ml/min)	
Urine microscopy	Protein + red cells nil	
Urine albumin/creatinine	133.62 mg/g	<30
HbA1c	8.5%	

**Fig. 2:**
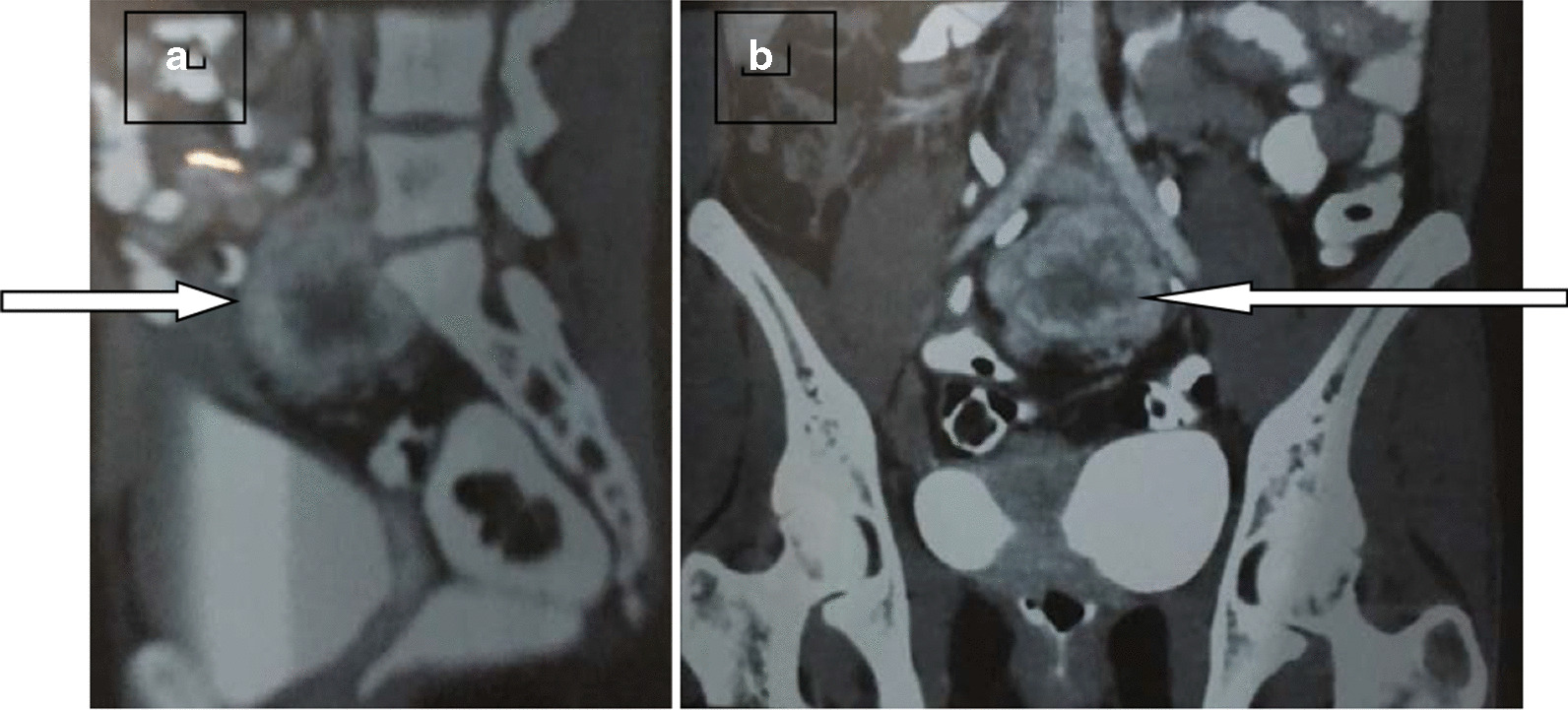
Computed tomography of pelvis with sagittal (**A**) and coronal sections (**B**) arrows showing well-defined soft tissue density measuring 5.1 × 5.2 × 4.7 cm with central necrosis

Following the biochemical confirmation of catecholamine-secreting tumor, our aim was to localize the problem. Since USS abdomen in 2012, which was done for dating scan, showed a 4.8 × 4.6 cm pelvic mass (Fig. 1A), we repeated her USS, which showed a well-defined, hypoechoic lesion at the fundus of the uterus measuring 5.5 × 4.5 cm with significant vascularity (Fig. 1B). Computed tomography (CT) showed well-defined soft-tissue density measuring 5.1 × 5.2 × 4.7 cm with central necrosis (Fig. 2A, B). Metaiodobenzylguanidine (MIBG) is indicated in patients with large pheochromocytoma (increased risk of malignancy) or paraganglioma (increased risk of malignancy and multiple tumor) [4, 5]. However, we could not proceed with it because of unavailability.

## Management and follow-up

Enalapril and hydrochlorothiazide were stopped, nifedipine continued, and prazosin started to target preoperative seated blood pressure target <130/80 mmHg and standing systolic blood pressure < 90 mmHg. Since her seated heart rate target of 60–70 beats per minute and standing heart rate target of 70–80 beats per minute were not achieved, she was started on metoprolol to control heart rate. She was advised to consume a high-sodium diet (> 5000 mg/day) and adequate fluid intake to increase treatment-induced volume contraction. Continuous administration of 2 liters of saline was given in the evening before surgery. She underwent excision of paraganglioma without any intraoperative hypertensive or hypotensive crises. Despite adequate control of fluid management postoperatively, she developed low blood pressure, which responded to fluid resuscitation.

**Fig. 3:**
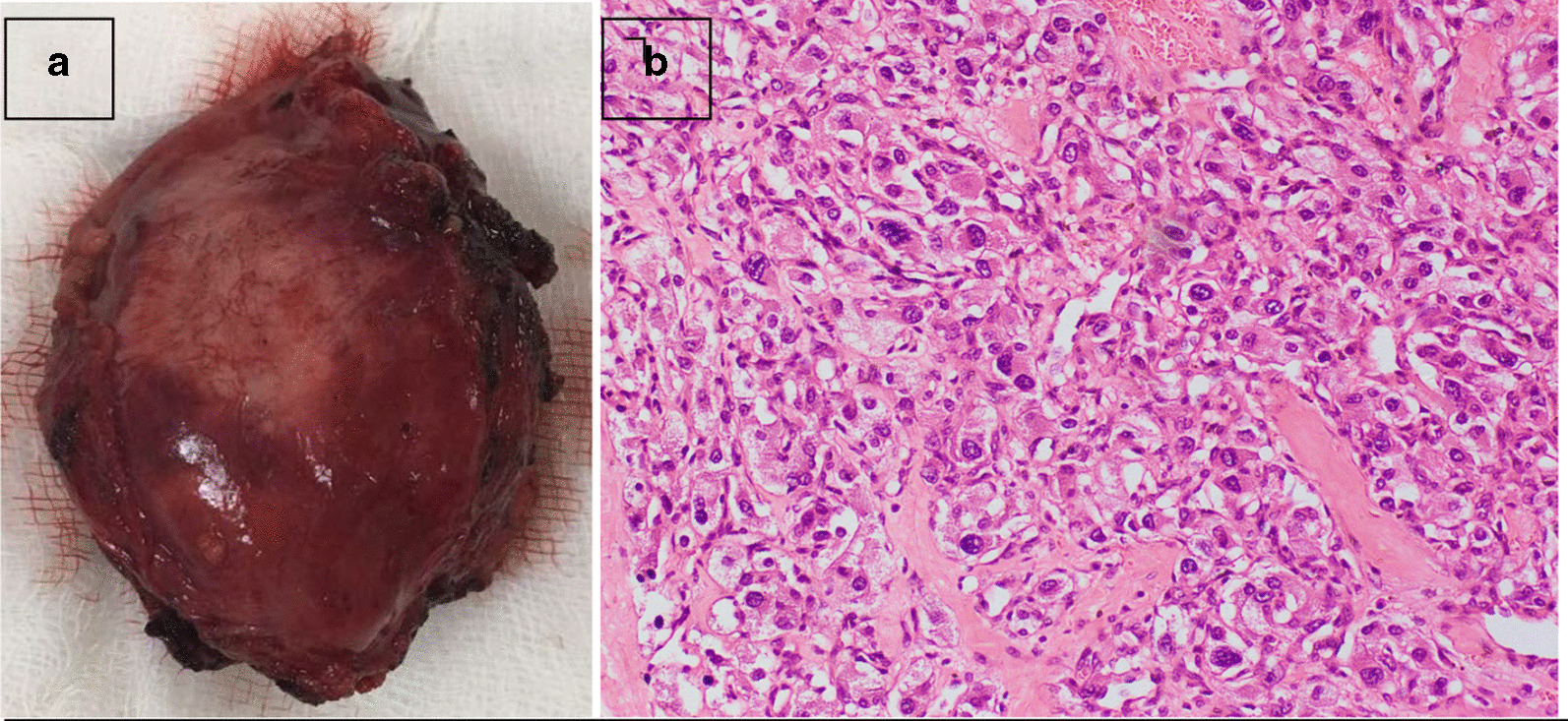
**A** Macroscopically: a partially circumscribed mass of tissue 7 × 4.5 × 4 cm^3^. **B** Microscopically, with hematoxylin and eosin staining: composed of polygonal cells arranged as compact nests (zellballen) surrounded by a flattened layer of sustentacular cells marked

Histology confirmed it was a paraganglioma (Fig. 3A shows macroscopic appearance, and 3B shows histology). One month postoperation, her chromogranin level was 16.03 ng/ml (1–76.3) and 24-hour urinary metanephrine was less than 1 mg/24 hours. Her blood sugar remained normal without any hypoglycemic agents, there was resolution of proteinuria (urine albumin/creatinine 9.54 mg/g), and platelet count dropped to 474,000 µl.

## Discussion

In our patient, secondary hypertension during pregnancy, diabetes mellitus, proteinuria, and thrombocytosis was due to pelvic paraganglioma. Her worsening or development of hypertension during pregnancy could be due to compression of the tumor by the gravid uterus. Her uncontrolled hypertension and necessity for emergency caesarean section following the start of labetalol can be explained by unopposed alpha activation. The presence of diabetes in a lean subject with body mass index (BMI) 20 kg/m^2^ should be a clue to think away from gestational diabetes mellitus or type 2 diabetes mellitus. However, both gestational diabetes mellitus and type 2 diabetes mellitus are highly prevalent among South Asians.

Results of a systematic review showed 143 cases of chromaffin cell tumors in pregnancy, with 112 adrenal pheochromocytomas, 28 extraadrenal paragangliomas, and three patients who had synchronous pheochromocytomas and paragangliomas [2]. The overall maternal mortality rate was 9.8% in pheochromocytomas and 3.6% in paragangliomas. Fetal mortality in women with pheochromocytomas was 16% compared with 12% for those with paragangliomas. The diagnosis was made antenatally in 84% of patients with paragangliomas and in 80.3% of those with pheochromocytomas. Hypertensive crises were lower in functional paraganglioma than in pheochromocytoma [6].

Proteinuria and diabetes mellitus can be a clinical feature in paraganglioma [7, 8]. Literature review did not identify any cases of thrombocytosis related to paraganglioma. However, reactive thrombocytosis in pheochromocytoma was restricted to few case reports [9–13].

Magnetic resonance imaging (MRI) without gadolinium is the imaging test of choice in pregnant women. Stimulation tests and metaiodobenzylguanidine (MIBG) scintigraphy are not considered safe for pregnant women [14]. Selection of antihypertensives to control blood pressure, mode of delivery, and type of anesthesia are important in patients with paraganglioma. Control of blood pressure in pregnancy should be balanced between placental compromise and adequate catecholamine blockade. Paraganglioma patients are prone to having labile blood pressure, placental abruption, and fetal loss. Choice of α blockade should be carefully considered. Phenoxybenzamine irreversibly block α receptors, has a long half-life, and crosses the placenta. In some cases, it has been associated with neonatal respiratory depression and hypotension. Doxazosin is a competitive α1 receptor blocker. Prazosin is an α blocker with a short half-life and needs multiple daily dosing. Vaginal delivery is avoided since it precipitates hypertensive crises during active labor. Delivery can be performed under spinal anesthesia since it may have the theoretical advantage of reducing neural stimulation to the adrenal glands and sympathetic chain if the block is high enough [2, 6, 15].

The possible reasons for missing paraganglioma in our case were lack of knowledge, low level of suspicion, and unavailability of expert at the center where the patient was managed initially. Selection of antihypertensives to control blood pressure, mode of delivery, and type of anesthesia are important in patients with paraganglioma.

## Conclusion

Paraganglioma is a very rare cause of pregnancy-induced hypertension, but it is associated with significant maternal and fetal mortality. Appropriate clinical suspicion, prompt diagnosis, and a multidisciplinary approach with careful monitoring are of paramount importance for a better outcome. This is the first case describing thrombocytosis due to paraganglioma, which became near normal after successful removal of the paraganglioma. Proteinuria is an unusual association with paraganglioma.

## Data Availability

Databases and all relevant raw data is available.
